# Association between Chronic Knee Pain and Psychological Stress in Those over 50 Years of Age: A Nationwide Cross-Sectional Study Based on the Sixth Korea National Health and Nutrition Examination Survey (KNHANES 2013–2015)

**DOI:** 10.3390/ijerph18189771

**Published:** 2021-09-16

**Authors:** Sangun Nah, Seong-San Park, Sungwoo Choi, Hae-Dong Jang, Ji-Eun Moon, Sangsoo Han

**Affiliations:** 1 Department of Emergency Medicine, Soonchunhyang University Bucheon Hospital, Bucheon 14584, Korea; potter325@naver.com (S.N.); csw3613@naver.com (S.C.); 2 Department of Orthopedic Surgery, Soonchunhyang University Bucheon Hospital, Bucheon 14584, Korea; sungsan5050@naver.com (S.-S.P.); khaki00@schmc.ac.kr (H.-D.J.); 3 Clinical Trial Center, Department of Biostatistics, Soonchunhyang University Bucheon Hospital, Bucheon 14584, Korea; moon6188@schmc.ac.kr

**Keywords:** knee joint, psychological distress, cross-sectional study

## Abstract

Chronic knee pain (CKP) can degrade the quality of life and cause dysfunction, resulting in the loss of independence. Psychological stress not only affects physical and mental health but is also a risk factor for CKP. In this cross-sectional study, we analyzed data from the sixth Korea National Health and Nutrition Examination Survey (2013–2015), and investigated the association between CKP and psychological stress of the Korean general population. The CKP status was determined based on survey responses of self-reported knee pain lasting for more than 30 days during the last 3 months. Psychological stress was classified as none, mild, moderate, or severe. The association between CKP and psychological stress was analyzed using multiple logistic regression analysis considering co-variables and demographic data. Logistic regression analysis adjusting for co-variables indicated that the risk of CKP increased with an increasing degree of stress, from mild (OR = 1.65, 95% CI 1.35–2.03, *p* < 0.001) to moderate (OR = 2.00, 95% CI 1.56–2.57, *p* < 0.001) and severe (OR = 3.02, 95% CI 2.08–4.37, *p* < 0.001). A significant association between the risk of CKP and psychological stress was identified. Therefore, when evaluating patients with CKP, it may be helpful for clinicians to check the degree of stress.

## 1. Introduction

Chronic knee pain (CKP) is a common symptom in people ≥ 50 years of age and may limit daily activities such as walking or stair climbing [1]. CKP can cause: direct costs, such as treatment and long-term care; indirect costs, such as reduced productivity and employment; intangible costs, such as pain, reduced social participation, and activity limitations [2]. In addition, CKP and disability caused by degenerative changes of cartilage in the elderly can lower the quality of life and cause dysfunction, resulting in the loss of individual independence [3].

The major cause of knee pain is osteoarthritis, and approximately half of those aged ≥50 years complain of knee pain, whereas 25% suffer from CKP [4,5]. Other risk factors for knee pain include a high body mass index (BMI), old age, a previous knee injury, female sex, and work that puts strain on the knee [6,7]. Moreover, socioeconomic status such as low education level is reported as a risk factor for knee osteoarthritis [8]. Psychosocial factors such as stress, anxiety, and depression have been reported to be related to the risk of CKP [9,10]. These psychosocial factors not only cause and exacerbate pain themselves but can also contribute to the process of converting acute pain into chronic pain; negative cognition and poor coping strategies, such as fear-avoidance and catastrophizing, may be involved [11,12,13]. Psychological stress has many effects on the physical and mental health of individuals, including diabetes mellitus, obesity, poor sleep quality, cortisol secretion, and depression [14]. However, because psychological stress is a subjective factor, there may be differences in the degree of stress felt by each individual, and the individual’s physiological response to stress may differ, so it is necessary to classify the degree of stress and determine its relationship with CKP [15].

Although many previous studies have investigated the association between psychological stress and CKP, there have been few based on the general population [16,17,18,19]. Therefore, this study analyzed the association between psychological stress and CKP in the Korean general population aged ≥50 years.

## 2. Materials and Methods

### 2.1. Study Design and Setting

This was a cross-sectional study to investigate the association between CKP and psychological stress of the Korean general population. We analyzed data from the Korean National Health and Nutrition Examination Survey (KNHANES) version VI conducted in 2013 (VI-1), 2014 (VI-2), and 2015 (VI-3). KNHANES is a survey conducted annually since 1998 by the Korea Centers for Disease Control and Prevention (KCDC) to investigate the South Korean population’s health and nutritional status. This survey was conducted with approximately 8000–10,000 participants selected using multistage, clustered, stratified, and random sampling according to their age, region, and sex. In total, 192 primary sampling units were proportionally assigned to represent the South Korean population, and about 4000 households were surveyed annually; each primary sampling unit was selected based on administrative districts and housing types. Because of the random nature of sampling, different participants were selected each year, and the same participants were not monitored. KNHANES is conducted by experienced medical staff and interviewers and consists of three parts: a health survey, a physical examination, and a nutrition survey [20]. We analyzed participants enrolled in KNHANES VI-1, 2, 3 (2013–2015), and excluded the following individuals: (1) under 50 years (KNHANES VI-1, 2, 3 did not investigate CKP in participants < 50 years old), (2) not having answered the CKP test questionnaire, and (3) not having answered the psychological stress questionnaire.

### 2.2. Definition of CKP and Psychological Stress

Participants were asked “Did you experience knee-joint pain for more than 30 days in the past 3 months?” Those who answered “yes” were considered to have CKP and were included in our study.

For evaluating psychological stress, participants were asked “How much stress do you feel in your daily life?” Answers were classified according to degree of stress as follows: none (“hardly any”), mild (“a little”), moderate (“a lot”), and severe (“very much” (Suggest using “an extremely high level”)).

### 2.3. Patient Demographics and Co-Variables

Through questionnaires and interviews, data on the demographics, socioeconomic status, lifestyle habits, and comorbidities of the participants were acquired. Waist circumference (cm) was measured at the narrowest part between the iliac crest and the lower rib cage margin. The body mass index was calculated by dividing each participant’s weight (kg) by the square of their height (m^2^), and the participants were classified into the following three groups: underweight (BMI < 18.5 kg/m^2^), normal (BMI = 18.5–24.9 kg/m^2^), or obese (BMI ≥ 25.0 kg/m^2^) [21]. The duration of sleep was investigated using the following question: “How long do you sleep per day?” Smoking status was recorded as non/ex-smoker or current smoker. Alcohol consumption was classified into the following four categories: none, ≤1 drink/month, 2 drinks/month to 3 drinks/week, or ≥4 drinks/week. The level of education was classified into the following four categories: ≤6 years (elementary school), 7–9 years (middle school), 10–12 years (high school), or ≥13 years (college or university). Regarding occupation status, participants were classified as unemployed (including housewives, students, etc.) or working in an office; sales and services; agriculture, forestry, and fisheries; or machine fitting and simple labor. The household income level (total monthly household income) was grouped into quartiles. Marital status was classified into the following five categories: single, married, separated, separated by death, and divorced. Physical activity was defined as either medium-intensity aerobic exercise of at least 2.5 h per week, high-intensity aerobic exercise of at least 1.25 h per week, or a mixture of medium- and high-intensity aerobic exercise performed for longer than stated above [22]. In addition, it was noted whether participants had ever been diagnosed with comorbidities such as hypertension, dyslipidemia, stroke, myocardial infarction, angina, arthritis, asthma, diabetes mellitus, depression, and/or malignancy.

### 2.4. Statistical Analyses

According to the presence of CKP, participants were divided into two groups, and general characteristics were compared between the groups. The chi-squared test was used to compare categorical variables, and Student’s *t*-test was used to compare continuous variables. Multiple logistic regression analysis was used to analyze the association between CKP and psychological stress. Odds ratios (ORs) were calculated with 95% confidence intervals (CIs). We confirmed the effects of co-variables using three multiple logistic regression models. Model 1 was unadjusted, model 2 was adjusted for age and sex, and model 3 was adjusted for age, sex, and other factors including waist circumference, obesity, the duration of sleep, smoking status, education level, alcohol consumption, household income, marital status, occupation, physical activity level, and comorbidities. IBM SPSS Statistics software (ver. 26.0; IBM Corp., Armonk, NY, USA) was used for statistical analyses. *p*-values < 0.05 were considered to indicate statistical significance, and bias was prevented using sampling weights.

## 3. Results

**Figure 1:**
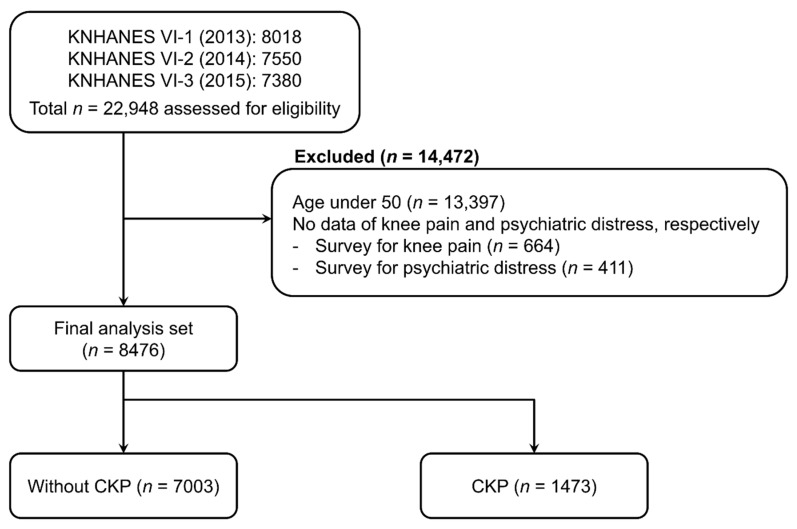
Flow chart of the study participants (Korea National Health and Nutrition Examination Survey [KNHANES], 2013–2015). Abbreviation: CKP, chronic knee pain.

A total of 22,948 people participated in KNHANES VI-1, 2, 3. Among them, 13,397 were aged < 50 years, 664 did not respond to the question about CKP, and 411 did not respond to the question about the degree of stress; therefore, these respondents were excluded from our study. Thus, 8476 participants were included in the final analysis; 7003 (82.6%) reported that they had no CKP, whereas 1473 (17.4%) reported that they had CKP (Figure 1).

### 3.1. Clinical Characteristics of Participants According to Presence of CKP

In the groups with and without CKP, 243 (16.5%) and 1811 (25.9%) participants reported experiencing no stress, 762 (51.7%) and 3958 (56.5%) reported mild stress, 356 (24.2%) and 988 (14.1%) reported moderate stress, and 112 (7.6%) and 246 (3.5%) reported severe stress (*p* < 0.001), respectively. There were significant differences between the two groups in terms of age, sex, obesity, the duration of sleep, alcohol consumption, education level, occupation, household income, and physical activity level (Table 1). The results of the general characteristics of participants according to degree of stress is presented in Supplementary Table S1.

### 3.2. Association between CKP and the Degree of Stress

**Figure 2:**
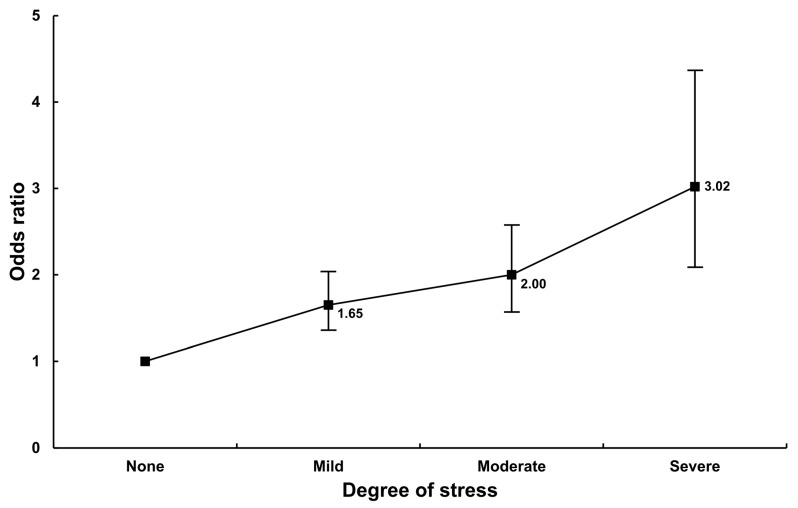
Association between CKP and psychological stress. The model was adjusted for age, sex, and other factors including obesity, the duration of sleep, smoking status, education level, alcohol consumption, household income, occupation, physical activity level, and comorbidities.

Multiple logistic regression analysis was used to analyze the association between CKP and the degree of stress (Table 2). In model 1 (not adjusted for any variables) and model 2 (adjusted for age and sex), the OR increased as the degree of stress increased. In model 3 (adjusted for all variables), the OR also increased as the degree of stress increased (mild: OR = 1.65, 95% CI 1.35–2.03, *p* < 0.001; moderate: OR = 2.00, 95% CI 1.56–2.57, *p* < 0.001; severe: OR = 3.02, 95% CI 2.08–4.37, *p* < 0.001) (Figure 2).

## 4. Discussion

By analyzing KNHANES data representing the Korean general population, our study confirmed the association between CKP and self-reported degree of stress. The most important finding is that, after adjusting for all co-variables, increasing stress was associated with increased risk of CKP. In particular, severe stress was associated with the highest risk of CKP (OR = 3.02; 95% CI, 2.08–4.37, *p* < 0.001).

Our findings were similar to those of previous studies that investigated the association between psychological stress and CKP [17,19]. Jones et al. conducted a 2-year follow-up study to investigate the risk factor of knee pain among 108 young, newly employed workers who had no knee pain and reported a significant correlation between knee pain and psychological distress [17]. Carotti et al. conducted a cross-sectional study in 149 patients with knee osteoarthritis and reported that a high level of psychological distress was associated with knee pain [19]. However, most studies did not subdivide psychological stress by intensity and simply combined stress levels to confirm the association with knee pain [17,19]. In addition, the results of these studies are not necessarily representative because research subjects were from specific occupational groups or were comprised mostly of women. Therefore, caution is needed when applying their findings to the general population. However, our study used survey data from the general population and applied multistage, clustered, stratified, and random sampling to obtain representativeness. In addition, by subdividing self-reported stress into four levels, the relationship between CKP and psychological stress was made clearer than in other studies.

**Figure 3:**
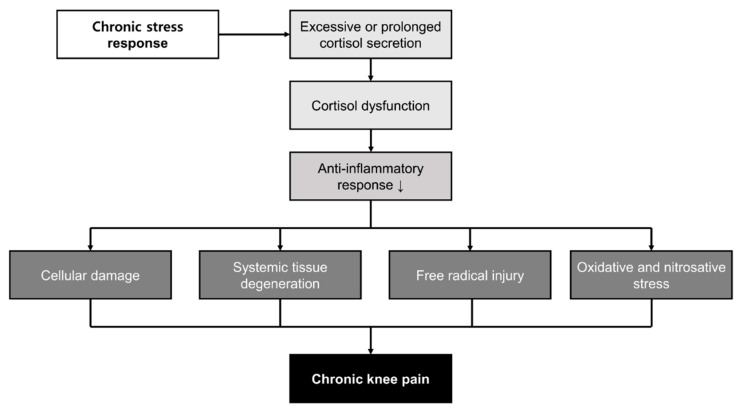
Hypothesis underlying the association between chronic knee pain and psychological stress. **Note:** “↓” means decreased.

We found that as the degree of stress increased, the OR for the risk of CKP increased. Stress is inevitable in everyday life, and humans have the ability to endure short-term stress; however, chronic stress can cause neuroendocrine changes in humans, resulting in chronic pain [23,24]. The stress response induces the secretion of sympathetic catecholamines (epinephrine and norepinephrine) and neuroendocrine hormones (cortisol) [24]. Under stress, the amygdala activates the hypothalamic–pituitary–adrenal (HPA) axis and causes the hypothalamus to secrete corticotropin-releasing hormone [25]. This hormone stimulates the secretion of adrenocorticotropic hormone in the anterior pituitary again and, consequently, stimulates the secretion of cortisol in the adrenal cortex [26]. Cortisol, which is regulated by the HPA axis and increases during stress responses, is important because it mobilizes glucose reserves and acts as a powerful anti-inflammatory agent [24]. Under short-term stress, the body can temporarily adapt to the secretion of cortisol, but continuous secretion of cortisol due to excessive or prolonged stress eventually induces cortisol dysfunction [25,26,27]. Cortisol dysfunction can cause symptoms such as bone and muscle breakdown, depression, and fatigue, while cellular damage, systemic tissue degeneration, free radical injury, and oxidative and nitrosative stress can occur due to the decrease in cortisol’s anti-inflammation effect [28,29,30] (Figure 3).

Our study had some limitations. First, because this study was a cross-sectional analysis based on a health survey conducted at the national level, a causal relationship between the degree of stress and CKP cannot be inferred. However, as the study was conducted using a multistage, clustered, and random sampling method, sampling error was minimized. Furthermore, the study was conducted on a general population, so the results are highly representative. Second, in the KNHANES dataset analyzed in this study, information on CKP was only available for participants ≥ 50 years old; thus, results for participants < 50 years old are unknown. Third, data on CKP and the degree of stress were obtained through a simple survey rather than a detailed quantitative method. CKP severity and the patterns of pain were not quantified, and psychological stress was not evaluated with an objective tool such as the perceived stress scale. Unfortunately, the KNHANES database does not contain information on scales related to stress. However, the association we found between self-reported stress and CKP is still meaningful. Fourth, there is a report that greater comorbidity burden may be able to impair the quality of life by worsening pain and physical function in people with chronic pain, but we did not analyze this relationship in our study [31]. Finally, the KNHANES data were obtained from the Korean general population, so our results are generalizable for Koreans. However, the association between CKP and the degree of stress may differ by race; therefore, to apply the results of our study worldwide, additional nationwide studies including various ethnicities will be needed. A large-scale, well-designed study (e.g., prospective cohort study) will be required in the future to overcome these limitations.

## 5. Conclusions

This cross-sectional study analyzed national health survey data and confirmed the association between CKP and psychological stress. Increasing psychological stress was associated with increased risk of CKP. In particular, the population with severe stress were in the highest risk of CKP. Therefore, when evaluating patients with CKP, it may be helpful for clinicians to check the degree of stress.

## Figures and Tables

**Table 1:** General characteristics of the study subjects according to presence of CKP.

Variables	Without CKP	With CKP	*p*-Value
	(*n* = 7003)	(*n* = 1473)	
Age, years	63.4 ± 8.9	67.0 ± 8.9	<0.001
Sex, *n* (%)			<0.001
Male	3249 (46.4)	352 (23.9)	
Female	3754 (53.6)	1121 (76.1)	
Waist circumference, cm	83.2 ± 9.1	84.8 ± 9.2	<0.001
Obesity status, *n* (%) ^¶^			<0.001
Underweight (BMI < 18.5)	188 (2.7)	26 (1.8)	
Normal (BMI = 18.5–24.9)	4377 (62.5)	809 (54.9)	
Obese (BMI ≥ 25)	2438 (34.8)	638 (43.3)	
Duration of sleep, h	6.6 ± 1.5	6.4 ± 1.7	<0.001
Smoking status, *n* (%)			<0.001
Non/ex-smoker	5914 (84.5)	1324 (89.9)	
Current smoker	1089 (15.6)	149 (10.1)	
Alcohol consumption, *n* (%)			<0.001
None	2658 (38.0)	743 (50.4)	
≤1 drink/month	1761 (25.2)	364 (24.7)	
2 drinks/month to 3 drinks/week	1994 (28.5)	277 (18.8)	
≥4 drinks/week	590 (8.4)	89 (6.0)	
Education level, *n* (%) ^‡^			<0.001
≤6 y	2659 (38.0)	965 (65.5)	
7–9 y	1229 (17.5)	238 (16.2)	
10–12 y	1939 (27.7)	213 (14.5)	
≥13 y	1176 (16.8)	57 (3.9)	
Occupation, *n* (%)			<0.001
Unemployed (student, housewife, etc.)	3368 (48.1)	905 (61.4)	
Office work	733 (10.5)	52 (3.5)	
Sales and services	768 (11.0)	110 (7.5)	
Agriculture, forestry, and fishery	1297 (18.5)	221 (15.0)	
Machine fitting and simple labor	837 (12.0)	185 (12.6)	
Household income, *n* (%) ^↕^			<0.001
Low	1866 (26.6)	667 (45.3)	
Low–moderate	1879 (26.8)	371 (25.2)	
Moderate–high	1593 (22.7)	238 (16.2)	
High	1665 (23.8)	197 (13.4)	
Marital status, *n* (%)			<0.001
Single	86 (1.2)	15 (1.0)	
Married	342 (4.9)	24 (1.6)	
Separated	1738 (24.8)	129 (8.8)	
Separated by death	3728 (53.2)	726 (49.3)	
Divorced	1109 (15.8)	579 (39.3)	
Degree of stress, *n* (%)			<0.001
None	1811 (25.9)	243 (16.5)	
Mild	3958 (56.5)	762 (51.7)	
Moderate	988 (14.1)	356 (24.2)	
Severe	246 (3.5)	112 (7.6)	
Physical activity, *n* (%)	1994 (30.1)	314 (21.4)	<0.001
Comorbidities, *n* (%)			
Hypertension	2519 (36.0)	710 (48.2)	<0.001
Dyslipidemia	1497 (21.4)	450 (30.6)	<0.001
Stroke	284 (4.1)	94 (6.4)	<0.001
Myocardial infarction	104 (1.5)	34 (2.3)	0.0311
Angina	211 (3.0)	82 (5.6)	<0.001
Arthritis	1057 (15.9)	883 (60.0)	<0.001
Asthma	205 (2.9)	103 (7.0)	<0.001
Diabetes mellitus	965 (13.8)	284 (19.3)	<0.001
Depression	35 (0.5)	16 (1.1)	0.014
Malignancy	200 (2.9)	49 (3.3)	0.375

Note: Values are presented as the means ± standard deviations for continuous variables or numbers (percentages) for categorical variables. ^¶^ The obesity status was determined based on the body mass index (BMI) as follows: underweight, BMI < 18.5 kg/m^2^; normal, BMI = 18.5–24.9 kg/m^2^; and obese, BMI ≥ 25.0 kg/m^2^. ^‡^ The educational level of each participant was assigned into one of the following four groups: ≤6 years (elementary school), 7−9 years (middle school), 10–12 years (high school), and ≥13 years (college or university). ^↕^ Household income level was assigned based on quartiles calculated from the total household monthly income of all participants.

**Table 2:** Association between CKP and psychological stress using multiple logistic regression.

	Model 1			Model 2			Model 3		
	OR	95% CI	*p*-Value	OR	95% CI	*p*-Value	OR	95% CI	*p*-Value
**Degree of stress**									
None	1			1			1		
Mild	1.38	1.16–1.64	<0.001	1.77	1.47–2.13	<0.001	1.65	1.35–2.03	<0.001
Moderate	2.45	1.98–3.02	<0.001	2.85	2.28–3.57	<0.001	2.00	1.56–2.57	<0.001
Severe	3.39	2.50–4.61	<0.001	4.17	3.00–5.80	<0.001	3.02	2.08–4.37	<0.001

## Data Availability

The data are available from the KCDC and Prevention database on the following webpage: https://knhanes.kdca.go.kr/knhanes/sub03/sub03_02_05.do (accessed on 3 June 2021). The data are available via this web page to anyone who meets the appropriate qualifications.

## Supplementary Materials

The following are available online at https://www.mdpi.com/article/10.3390/ijerph18189771/s1, Supplemental Table S1: General characteristics of the study subjects according to degree of stress.
