# Supplementary material for: Association between Chronic Knee Pain and Psychological Stress in Those over 50 Years of Age: A Nationwide Cross-Sectional Study Based on the Sixth Korea National Health and Nutrition Examination Survey (KNHANES 2013–2015)
Source: Int J Environ Res Public Health. 2021 Sep 16;18(18):9771. doi: 10.3390/ijerph18189771 (PMC8467605; doi:10.3390/ijerph18189771)
Supplement: Supplementary file 1 [file ijerph-18-09771-s001.zip › ijerph-1292566-supplementary.pdf]

Supplementary materials: Association between chronic knee pain and psychological stress in those over 50 years of age: a nationwide cross-sectional study based on the sixth Korea National Health and Nutrition Examination Survey (KNHANES 2013–2015)

Sangun Nah, Seong San Park, Sungwoo Choi, Hae-Dong Jang, Ji Eun Moon and Sangsoo Han

Table S1. General characteristics of the study subjects according to degree of stress

	**None** (*n* = 2054)	**Mild** (*n* = 4720)	**Moderate** (*n* = 1344)	**Severe** (*n* = 358)	*p*-value
Age, years	67.3 ± 9.1	62.9 ± 8.6	63.3 ± 9.2	63.1 ± 9.0	<0.001
Sex, *n* (%)					<0.001
Male	969 (47.2)	2063 (43.7)	444 (33.0)	125 (34.9)	
Female	1085 (52.8)	2657 (56.3)	900 (67.0)	233 (65.1)	
Waist circumference, cm	84.3 ± 9.3	83.4 ± 8.9	83.0 ± 9.3	83.3 ± 10.3	<0.001
Obesity status, *n* (%)[¶]					0.200
Underweight (BMI < 18.5)	55 (2.7)	105 (2.2)	39 (2.9)	15 (4.2)	
Normal (BMI = 18.5–24.9)	1235 (60.1)	2906 (61.6)	834 (62.1)	211 (58.9)	
Obese (BMI ≥ 25)	764 (37.2)	1709 (36.2)	471 (35.0)	132 (36.9)	
Duration of sleep, h	6.7 ± 1.6	6.6 ± 1.4	6.4 ± 1.6	6.2 ± 1.9	<0.001
Smoking status, *n* (%)					0.007
Non/ex-smoker	1792 (87.2)	4021 (85.2)	1135 (84.5)	290 (81.0)	
Current smoker	262 (12.8)	699 (14.8)	209 (15.6)	68 (19.0)	
Alcohol consumption, *n* (%)					<0.001
None	898 (43.7)	1757 (37.2)	581 (43.2)	165 (46.1)	
≤ 1 drink/month	474 (23.1)	1243 (26.3)	332 (24.7)	76 (21.2)	
2 drinks/month to 3 drinks/week	496 (24.2)	1362 (28.9)	334 (24.9)	79 (22.1)	
≥ 4 drinks/week	186 (9.1)	358 (7.6)	97 (7.2)	38 (10.6)	
Education level, *n* (%)[‡]					<0.001
≤ 6 y	981 (47.8)	1818 (38.5)	625 (46.5)	200 (55.9)	
7–9 y	336 (16.4)	870 (18.4)	208 (15.5)	53 (14.8)	
10–12 y	447 (21.8)	1325 (28.1)	313 (23.3)	67 (18.7)	
≥ 13 y	290 (14.1)	707 (15.0)	198 (14.7)	38 (10.6)	
Occupation, *n* (%)					<0.001
Unemployed (student, housewife, etc.)	1175 (57.2)	2237 (47.4)	659 (49.0)	202 (56.4)	
Office work	142 (6.9)	482 (10.2)	135 (10.0)	26 (7.3)	
Sales and services	141 (6.9)	526 (11.1)	174 (12.9)	37 (10.3)	
Agriculture, forestry, and fishery	349 (17.0)	894 (18.9)	219 (16.3)	56 (15.6)	
Machine fitting and simple labor	247 (12.0)	581 (12.3)	157 (11.7)	37 (10.3)	
Household income, *n* (%)[↕]					<0.001
Low	727 (35.4)	1197 (25.4)	468 (34.8)	141 (39.4)	
Low-moderate	553 (26.9)	1244 (26.4)	342 (25.4)	111 (31.0)	

Moderate-high	391 (19.0)	1125 (23.8)	257 (19.1)	58 (16.2)	
High	383 (18.6)	1154 (24.4)	277 (20.6)	48 (13.4)	
Marital status, *n* (%)					<0.001
Single	24 (1.2)	46 (1.0)	18 (1.3)	13 (3.6)	
Married	171 (8.3)	161 (3.4)	22 (1.6)	12 (3.4)	
Separated	572 (27.8)	988 (20.9)	214 (15.9)	36 (10.1)	
Separated by death	1007 (49.0)	2654 (56.2)	691 (51.4)	149 (41.6)	
Divorced	280 (13.6)	871 (18.5)	399 (29.7)	148 (41.3)	
Physical activity, *n* (%)	481 (24.4)	1420 (31.5)	329 (25.7)	78 (23.2)	<0.001
CKP, *n* (%)	243 (11.8)	762 (16.1)	356 (26.5)	112 (31.3)	<0.001
Comorbidities, *n* (%)					
Hypertension	869 (42.3)	1708 (36.2)	509 (37.9)	143 (39.9)	<0.001
Dyslipidemia	426 (20.7)	1097 (23.2)	324 (24.1)	100 (27.9)	0.008
Stroke	114 (5.6)	179 (3.8)	68 (5.1)	17 (4.8)	0.008
Myocardial infarction	32 (1.6)	76 (1.6)	24 (1.8)	6 (1.7)	0.963
Angina	58 (2.8)	154 (3.3)	57 (4.2)	24 (6.7)	<0.001
Arthritis	419 (21.2)	1048 (23.2)	370 (28.8)	103 (30.6)	<0.001
Asthma	81 (3.9)	150 (3.2)	58 (4.3)	19 (5.3)	0.046
Diabetes mellitus	360 (17.5)	618 (13.1)	208 (15.5)	63 (17.6)	<0.001
Depression	5 (0.2)	15 (0.32)	19 (1.4)	12 (3.4)	<0.001
Malignancy	66 (3.2)	131 (2.8)	40 (3.0)	12 (3.4)	0.752

Note: Values are presented as the means ± standard deviations for continuous variables or numbers (percentages) for categorical variables.

¶The obesity status was determined based on the body mass index (BMI) as follows: underweight, BMI < 18.5 kg/m^2; normal, BMI = 18.5–24.9 kg/m^2; and obese, BMI ≥ 25.0 kg/m^2.

‡The educational level of each participant was assigned into one of the following four groups: ≤ 6 years (elementary school), 7–9 years (middle school), 10–12 years (high school), and ≥ 13 years (college or university).

⌶Household income level was assigned based on quartiles calculated from the total household monthly income of all participants.
